# The Potential of Current Noninvasive Wearable Technology for the Monitoring of Physiological Signals in the Management of Type 1 Diabetes: Literature Survey

**DOI:** 10.2196/28901

**Published:** 2022-04-08

**Authors:** Elena Daskalaki, Anne Parkinson, Nicola Brew-Sam, Md Zakir Hossain, David O'Neal, Christopher J Nolan, Hanna Suominen

**Affiliations:** 1 School of Computing College of Engineering and Computer Science The Australian National University Canberra Australia; 2 Department of Health Services Research and Policy, Research School of Population Health College of Health and Medicine The Australian National University Canberra Australia; 3 School of Biology College of Science The Australian National University Canberra Australia; 4 Bioprediction Activity Commonwealth Industrial and Scientific Research Organisation Canberra Australia; 5 Department of Medicine University of Melbourne Melbourne Australia; 6 Department of Endocrinology and Diabetes St Vincent’s Hospital Melbourne Melbourne Australia; 7 Australian National University Medical School and John Curtin School of Medical Research College of Health and Medicine The Autralian National University Canberra Australia; 8 Department of Diabetes and Endocrinology The Canberra Hospital Canberra Australia; 9 Data61 Commonwealth Industrial and Scientific Research Organisation Canberra Australia; 10 Department of Computing University of Turku Turku Finland

**Keywords:** type 1 diabetes, wearable sensors, big data, consumer health informatics, mobile health, survey

## Abstract

**Background:**

Monitoring glucose and other parameters in persons with type 1 diabetes (T1D) can enhance acute glycemic management and the diagnosis of long-term complications of the disease. For most persons living with T1D, the determination of insulin delivery is based on a single measured parameter—glucose. To date, wearable sensors exist that enable the seamless, noninvasive, and low-cost monitoring of multiple physiological parameters.

**Objective:**

The objective of this literature survey is to explore whether some of the physiological parameters that can be monitored with noninvasive, wearable sensors may be used to enhance T1D management.

**Methods:**

A list of physiological parameters, which can be monitored by using wearable sensors available in 2020, was compiled by a thorough review of the devices available in the market. A literature survey was performed using search terms related to T1D combined with the identified physiological parameters. The selected publications were restricted to human studies, which had at least their abstracts available. The PubMed and Scopus databases were interrogated. In total, 77 articles were retained and analyzed based on the following two axes: the reported relations between these parameters and T1D, which were found by comparing persons with T1D and healthy control participants, and the potential areas for T1D enhancement via the further analysis of the found relationships in studies working within T1D cohorts.

**Results:**

On the basis of our search methodology, 626 articles were returned, and after applying our exclusion criteria, 77 (12.3%) articles were retained. Physiological parameters with potential for monitoring by using noninvasive wearable devices in persons with T1D included those related to cardiac autonomic function, cardiorespiratory control balance and fitness, sudomotor function, and skin temperature. Cardiac autonomic function measures, particularly the indices of heart rate and heart rate variability, have been shown to be valuable in diagnosing and monitoring cardiac autonomic neuropathy and, potentially, predicting and detecting hypoglycemia. All identified physiological parameters were shown to be associated with some aspects of diabetes complications, such as retinopathy, neuropathy, and nephropathy, as well as macrovascular disease, with capacity for early risk prediction. However, although they can be monitored by available wearable sensors, most studies have yet to adopt them, as opposed to using more conventional devices.

**Conclusions:**

Wearable sensors have the potential to augment T1D sensing with additional, informative biomarkers, which can be monitored noninvasively, seamlessly, and continuously. However, significant challenges associated with measurement accuracy, removal of noise and motion artifacts, and smart decision-making exist. Consequently, research should focus on harvesting the information hidden in the complex data generated by wearable sensors and on developing models and smart decision strategies to optimize the incorporation of these novel inputs into T1D interventions.

## Introduction

### Type 1 Diabetes

In healthy individuals, glucose levels are maintained within tight upper and lower bounds because of a complex physiological closed-loop regulatory process based on the accurate and timely secretion of insulin and glucagon into the portal vein by pancreatic islet cells [1]. To achieve this, the human body contains numerous sensory mechanisms that track and even anticipate fluctuations in glucose owing to meal intake, exercise, and other factors. This information is used to estimate the optimal secretion of insulin as well as other hormones, such as incretins, glucagon, and adrenaline, which play important modulating roles [2].

In persons with type 1 diabetes (T1D), insulin secretion by the pancreas is absent because of the autoimmune destruction of the pancreatic beta cells, breaking the normal closed-loop regulation process [3]. The absence of insulin results in the inability to metabolize glucose and an unregulated catabolic state leading to hyperglycemia and ketoacidosis, a recognized complication of diabetes [4]. Persons living with T1D are dependent on exogenous insulin (usually by subcutaneous administration) to regulate their glucose levels [5].

Poor glucose control leads to both acute and chronic complications, which may be life-threatening [6]. One common acute complication is hypoglycemia, a state of low blood glucose (BG) concentration that results from excessive insulin administration. Hypoglycemia can lead to loss of consciousness, coma, and even death [7]. Those who develop impaired awareness to hypoglycemia (IAH), a condition in which the individual does not experience the usual early warning symptoms of low BG, are at a much higher risk of severe hypoglycemic events [8].

On the other hand, chronic hyperglycemia leads to long-term complications, one of the most common being cardiac autonomic neuropathy (CAN). The reported prevalence of CAN in persons with T1D spans a very wide range, indicatively 17%-90%, depending on the criteria used for its diagnosis and the population studied [9]. CAN results in impaired cardiovascular autonomic control as a consequence of autonomic nerve neuronal metabolic and ischemic damage. Apart from CAN, T1D is linked to other long-term complications such as peripheral neuropathy, retinopathy, nephropathy, and atherosclerotic vascular disease [6].

Maintaining glucose levels within a healthy range in T1D represents a therapeutic challenge, and the accurate estimation of an individual’s insulin requirements is key to achieving this goal [10,11]. Daily glucose management places a significant physical and cognitive burden on the persons living with T1D and their families. As part of ambulatory care, long and rapid acting insulins are administered using insulin injections (insulin pens) or rapid acting insulin is administered continuously and subcutaneously by insulin infusion pumps. Both insulin pumps and injections may be used in combination with continuous glucose monitors (CGMs). Insulin pumps integrated with CGMs are referred to as sensor-augmented pumps and their use has been shown to improve glycemic control [12,13]. More recently, control algorithms have been incorporated to automate basal insulin delivery; however, the user is still required to administer a bolus before meals. This treatment scheme is referred to as a hybrid closed-loop system. Hybrid closed-loop schemes have been shown to improve glucose control in comparison with insulin dosing entirely determined by the user [14] and have paved the way for the holy grail of T1D management, which is the development of fully closed-loop functionality or an artificial pancreas (AP) [15,16].

For optimal T1D management, accurate real-time sensing of key physiological parameters (ie, biomarkers) is essential. Sensing is crucial for daily acute glycemic management to ensure the correct estimation of insulin dose, as well as for the early recognition of long-term complications. To date, T1D sensing for acute glycemic management is focused on glucose monitoring, measured either by finger pricking or CGMs, which are associated with painful and sparse measurements or significant sensing lags, respectively [17,18]. However, monitoring of glucose alone has significant limitations in informing optimal insulin delivery, whether in open or closed-loop systems. In addition, monitoring of diabetes complications such as CAN is predominantly performed through infrequent assessments during clinic visits. Please refer to Multimedia Appendix 1 [9,17-23] for a more detailed discussion on the current status and challenges of sensing biomarkers of T1D. This survey, which reviewed 77 publications, investigated novel physiological parameters with the potential to be incorporated into T1D management systems and reduce the impact of T1D on quality of life, specifically on parameters that can be monitored with the wearable sensors available today.

### Wearable Sensors

**Figure 1:**
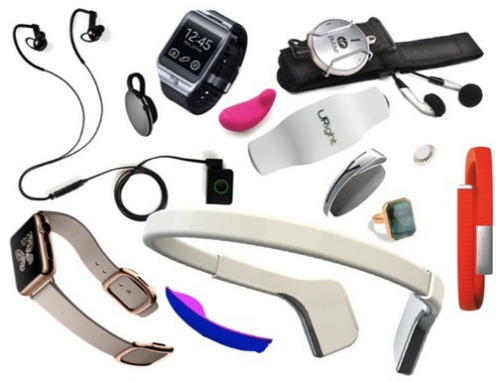
Different types of wearable technology by ForbesOste (license: CC BY-NC-ND 2.0).

Wearable sensors or wearables are becoming increasingly popular because they can provide seamless and continuous monitoring at low cost. Wearable sensors are available in various forms and shapes and can be worn at different body sites (Figure 1). Currently, wearables monitor physiological parameters, such as heart rate (HR), respiratory rate, oxygen saturation (SpO_2_), skin temperature (ST), electrochemical skin conductance (ESC), and galvanic skin response (GSR). The most recent devices provide electrocardiogram (ECG) monitoring, which carries rich information about features of the cardiac state, such as HR variability (HRV) and QT interval (time interval from the start of the Q-wave to the end of the T-wave in an ECG).

This offers new potential for wearable devices to move beyond their original purpose of fitness and wellness monitoring to that of continuous health care monitoring. Although not yet integrated into clinical practice, research community is currently investing in the development of medical applications using wearable sensors that can assist or complement routine medical procedures and disease monitoring practices. These efforts usually combine wearable sensors with advanced data processing methods such as machine learning (ML) algorithms to address the volume and complexity of the produced data (noise, motion artifacts, and gaps) and to build smart decision support or diagnostic systems (Multimedia Appendix 2 [24-37]).

Despite this recent move toward health care, the potential of wearables in T1D management has not been investigated much to date. The purpose of this survey was to explore the potential of monitoring physiological parameters with wearable sensors to assist in acute glycemic management and diagnosis and monitoring of complications in T1D. We devised a search and analysis framework to investigate the potential of *wearable, noninvasive sensors* (hereafter referred to as wearable sensors without explicitly repeating their noninvasive property). To the authors’ knowledge, a similar survey has not been conducted thus far. We hypothesized that the demonstration of strong links between physiological parameters measurable with wearable sensors and T1D, combined with their intrinsic advantages for use, will support and inspire developments and research focused on the enhancement of real-life T1D applications.

## Methods

### Research Question

The survey aimed to answer the following research question: *Do the existing wearable, noninvasive sensors have the potential to improve how T1D is monitored and managed?* We focused only on noninvasive wearable sensors. Minimally invasive wearables (eg, CGMs) or noninvasive portable sensors (eg, breath sensors) were outside the scope of the survey. We did not limit our search to studies that used wearable sensors but rather formulated our research question toward exploring the existence of clinical relationships between T1D and physiological parameters that could be (but not necessarily) monitored with the wearable, noninvasive technology available in the market today.

### Search Methodology

As a first step, a list of physiological parameters that could be monitored with wearable, noninvasive sensors available in 2020 was compiled, based on a review of the wearable devices available in the market today. These parameters were used in the search query combined with keywords related to T1D (Textboxes 1 and 2).

**Figure 2:**
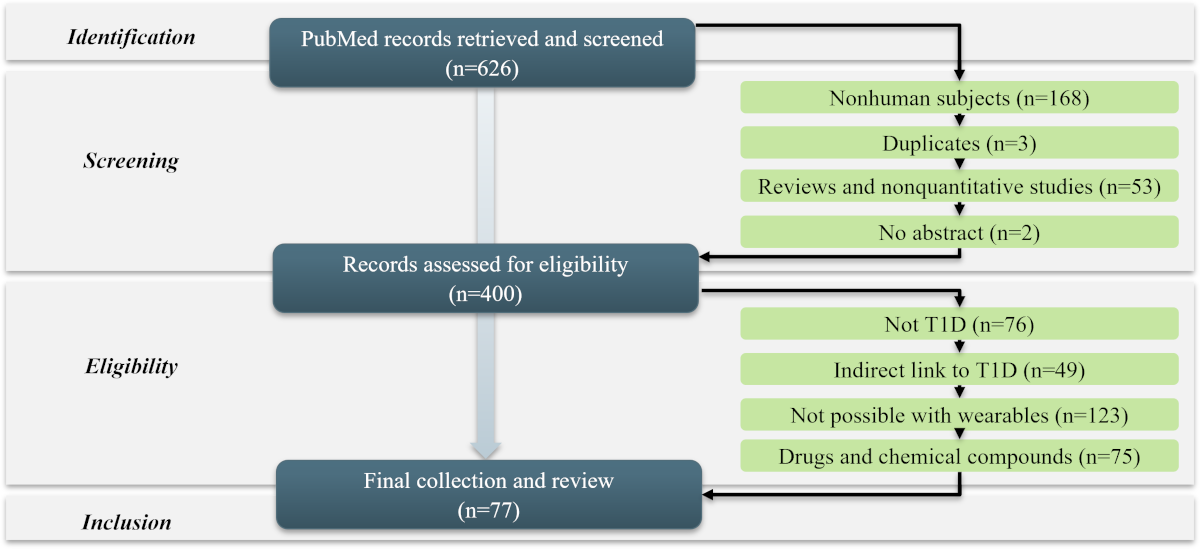
Article selection methodology according to PRISMA (Preferred Reporting Items for Systematic Reviews and Meta-Analyses) guidelines. T1D: type 1 diabetes.

Figure 2 illustrates the methodological steps we took to select the final set of articles, following the PRISMA (Preferred Reporting Items for Systematic Reviews and Meta-Analyses) guidelines [38]. The details of the search and selection methodology are described in Multimedia Appendix 3 [38]. Following these steps, 77 articles were retained for analysis.

**Search terms used for wearable-enabled physiological parameters.**

**Heart rate**

- Heart rate

**Heart rate variability**

- Heart rate variability

**Breath rate**

- Breath* rate
- Respirat* rate

**Breath rate variability**

- Respirat* variability
- Breath* rate variability

**Oxygen saturation**

- Oxygen saturation
- SpO_2_

**Body motion**

- Accelerometer*
- Gyroscope

**Skin properties**

- Galvanic skin response
- Skin conductance
- Skin impedance
- Skin temperature
- Sweat

**The final search query used.**

**Search query**

- Diabetes AND ("Type 1" OR "Type one" OR juvenile) AND ("heart rate" OR "heart rate variability" OR "respiration rate" OR "respiratory rate" OR "breath rate" OR "breathing rate" OR "respiration variability" OR "galvanic skin response" OR "skin conductance" OR "skin impedance" OR sweat OR accelerometer* OR gyroscope* OR "oxygen saturation" OR SpO_2_)

## Results

### Article Structure and Analysis

The 77 retained articles shared similar structures. Data were collected through a clinical protocol whereby each study collected two types of data: (1) phenotypic characteristics of the participants with T1D (eg, age, sex, duration of diabetes, and glucose and hemoglobin A_1c_ [HbA_1c_] levels), with or without the presence of nondiabetic control participants and (2) physiological function of interest (eg, cardiac autonomic function), with one or more physiological parameters measured (eg, HRV for autonomic function and ESC for sudomotor function). Through the extraction of features and data analysis, every study explored the relationship between T1D phenotypic characteristics and the physiological function of interest. All articles referred to clinical studies involving human participants. Although the articles did not necessarily use wearable devices in their methods, they all measured physiological parameters monitorable with commercially available wearable sensors, as guaranteed by our exclusion criteria (please refer to Multimedia Appendix 3).

Our analysis of the retained articles evolved along the axis of this survey’s research question: *Do the existing wearable, noninvasive sensors have the potential to improve how T1D is monitored and managed?* The surveyed studies were clustered into the following two broad categories: (1) those that performed a comparison between a cohort of persons with T1D and a matched group of healthy control participants and (2) those that explored relations within a cohort of persons with T1D. The first category informed about the physiological functions and parameters that were altered in persons living with T1D. The second category explored whether the monitoring of these parameters could benefit the management of T1D and its complications. All articles included in this survey are tabulated in Multimedia Appendix 4 [39-114].

### Comparison Between Persons With T1D and Healthy Cohorts

#### Overview

**Figure 3:**
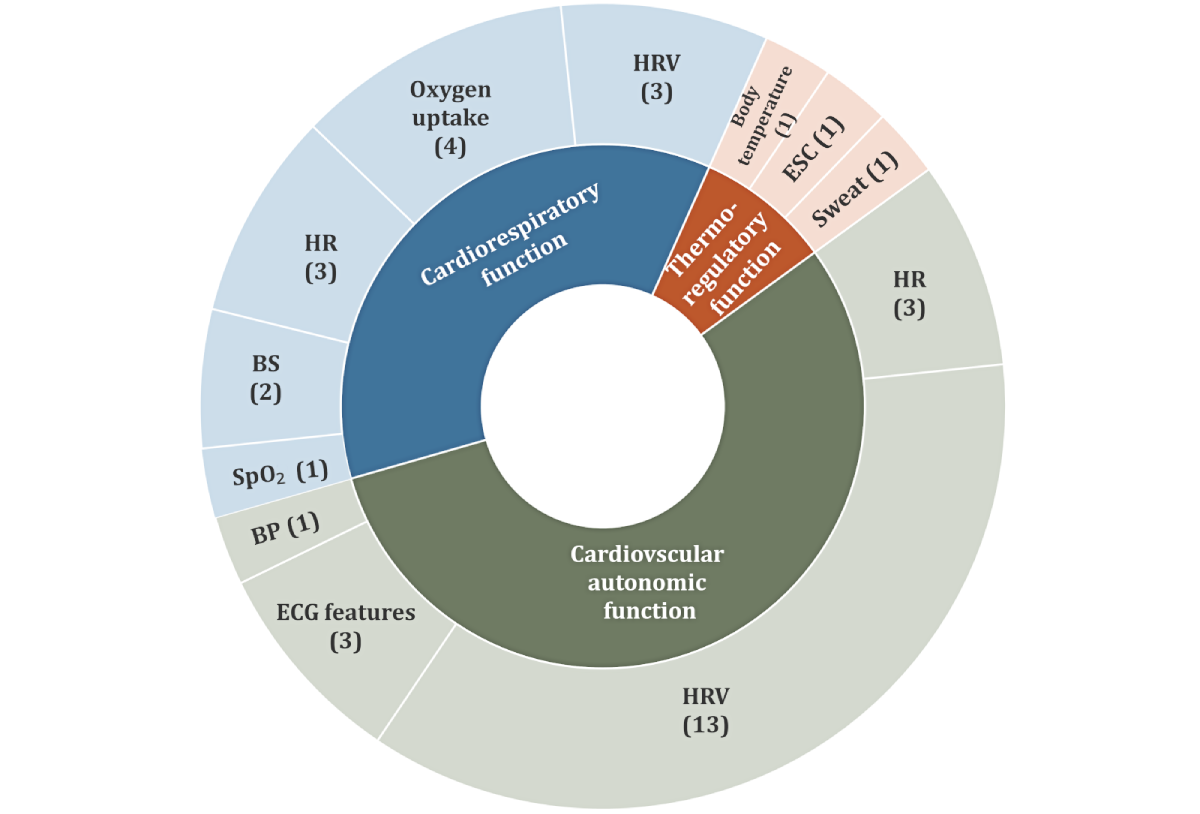
Physiological functions found that are affected by type 1 diabetes (T1D) and the number of relevant studies. This graph refers only to the studies that compared T1D with healthy cohorts. BP: blood pressure; BS: Baroreflex Sensitivity; ECG: electrocardiogram; ESC: electrochemical skin conductance; HR: heart rate; HRV: heart rate variability; SpO_2_: oxygen saturation.

The articles that compared people who were healthy and those with T1D revealed a wide spectrum of physiological functions or conditions that were affected by T1D (Figure 3). These predominantly included aspects of cardiovascular autonomic function, cardiorespiratory control balance, and thermal homeostasis.

#### Cardiovascular Autonomic Function

Many studies demonstrated that T1D impacted cardiac autonomic function by *reducing parasympathetic activity* through increased HR [39,40], reduced average HRV, and modified HRV features at rest or during cardiac autonomic reflex tests. The most explored HRV features were the following time- and frequency-domain features: SD of the normal-to-normal intervals, root mean square of the difference of successive intervals, high frequency (HF), low frequency (LF), and their ratio (LF/HF) [41-43]. Nonlinear features related to the complexity, dynamics, and chaotic components of HRV have also shown to be altered in persons with T1D. These include HRV randomness [44], symbolic indices [45], Katz fractal dimension [46], and geometric indices, such as the Poincare plot [47]. The effect of T1D on HRV was also demonstrated during exercise [48]. The overall HRV entropy during vigorous exercise was shown to be reduced in both healthy and T1D cohorts, although the attenuation in the T1D group was greater [49]. The impact on parasympathetic function was additionally demonstrated through the *reduction of the cardiac vagal tone*, which was shown to be associated with the presence of neuropathy [50]. *Cardiac depolarization and repolarization time intervals* were shown to increase in T1D demonstrated through modified ECG features such as increased HR-corrected QT (QT corrected [QTc]) interval [51,52] and more asymmetrical and flatter T-wave [53]. Table S1 in Multimedia Appendix 4 summarizes the methods and main findings of the above articles.

#### Cardiorespiratory Control Balance and Fitness

The impact of T1D on the *cardiorespiratory control balance and fitness* was assessed during cardiopulmonary exercise, as well as by cardiovascular (baroreflex) and respiratory (chemoreflex) response testing (Table S2 in Multimedia Appendix 4). Moser et al [54] demonstrated that there were clear differences in the HR response during cardiopulmonary exercise between persons with T1D and those in the control group. Cardiorespiratory control imbalance, with impaired *sensitivity to hypoxia* as evidenced by lower *resting SpO_2_* and *baroreflex sensitivity* and increased *chemoreflex sensitivity to hypercapnia*, was shown in persons with recent onset of T1D compared with control participants [55]. With respect to cardiorespiratory fitness, lower *peak oxygen uptake (VO_2_)* and *respiratory exchange ratios* were reported in persons with T1D compared with control participants [56]; however, Rohling et al [57] showed that cardiorespiratory function was preserved in T1D, whereas the maximum VO_2_ correlated with HRV time indices. Several studies demonstrated that persons with T1D who were well trained or well controlled could have similar cardiorespiratory fitness as the control participants [58-61].

#### Thermal Homeostasis

The *sudomotor function* is an activation of the sympathetic nervous system, controls the perspiration through sweat glands and heat loss via skin, and is associated with increased blood flow. This function, which is usually assessed by measuring the ESC or GSR, was shown to be lower in persons with T1D compared with healthy control participants [62]. Other aspects of thermal homeostasis affected in persons with T1D included the *sweat profile, body temperature*, and *ST during exercise* [49,63,64]. The methods and findings of the aforementioned studies are presented in Table S3 in Multimedia Appendix 4.

#### Summary

The aforementioned studies demonstrated that T1D affected several physiological functions by comparing T1D cohorts with healthy control participants under resting conditions or during physical activity. Most of the studies focused on aspects of cardiac autonomic function, which seems to be one of the first nonmetabolic physiological functions affected by T1D.

### Correlations Within a T1D Cohort

#### Overview

**Figure 4:**
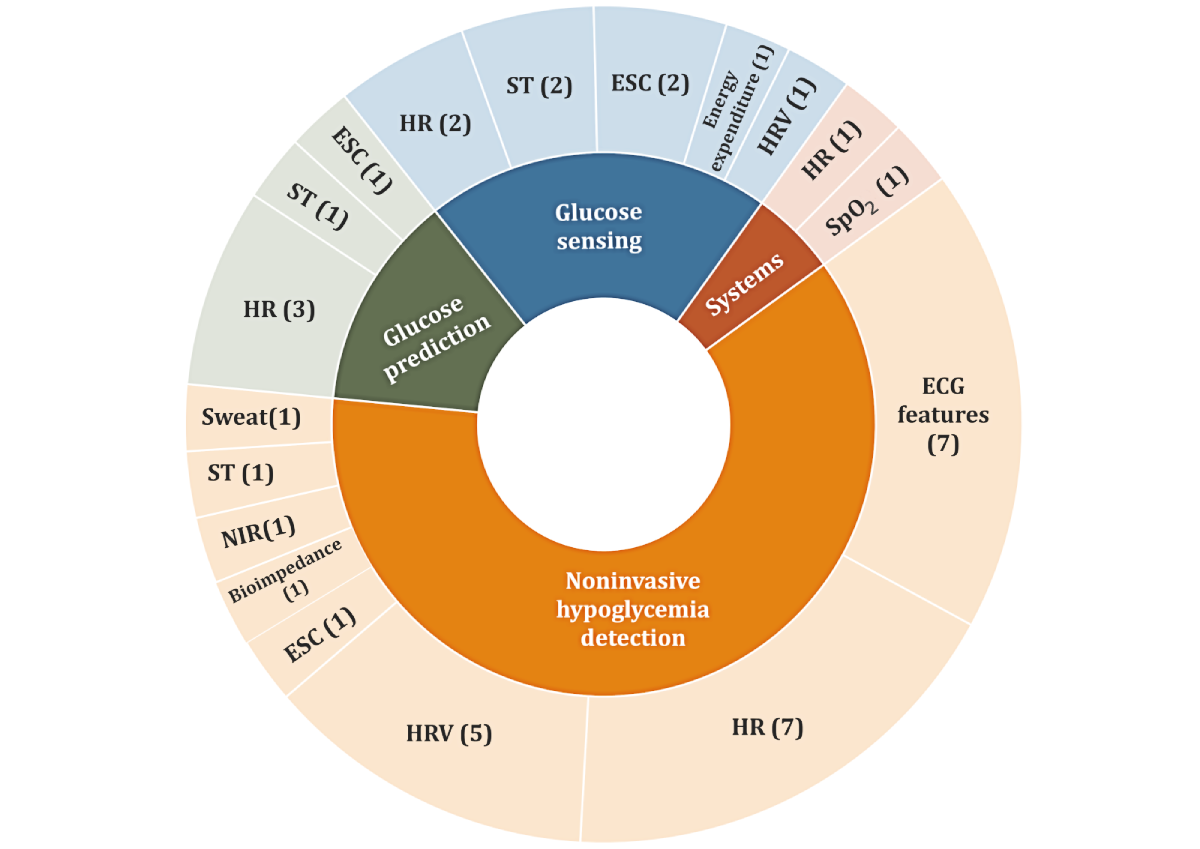
Identified areas of potential impact of wearable devices on the acute glycemic management of type 1 diabetes. The numbers indicate the number of relevant studies found for each physiological signal. ECG: electrocardiogram; ESC: electrochemical skin conductance; HR: heart rate; HRV: heart rate variability; NIR: near-infrared; SpO_2_: oxygen saturation; ST: skin temperature.

**Figure 5:**
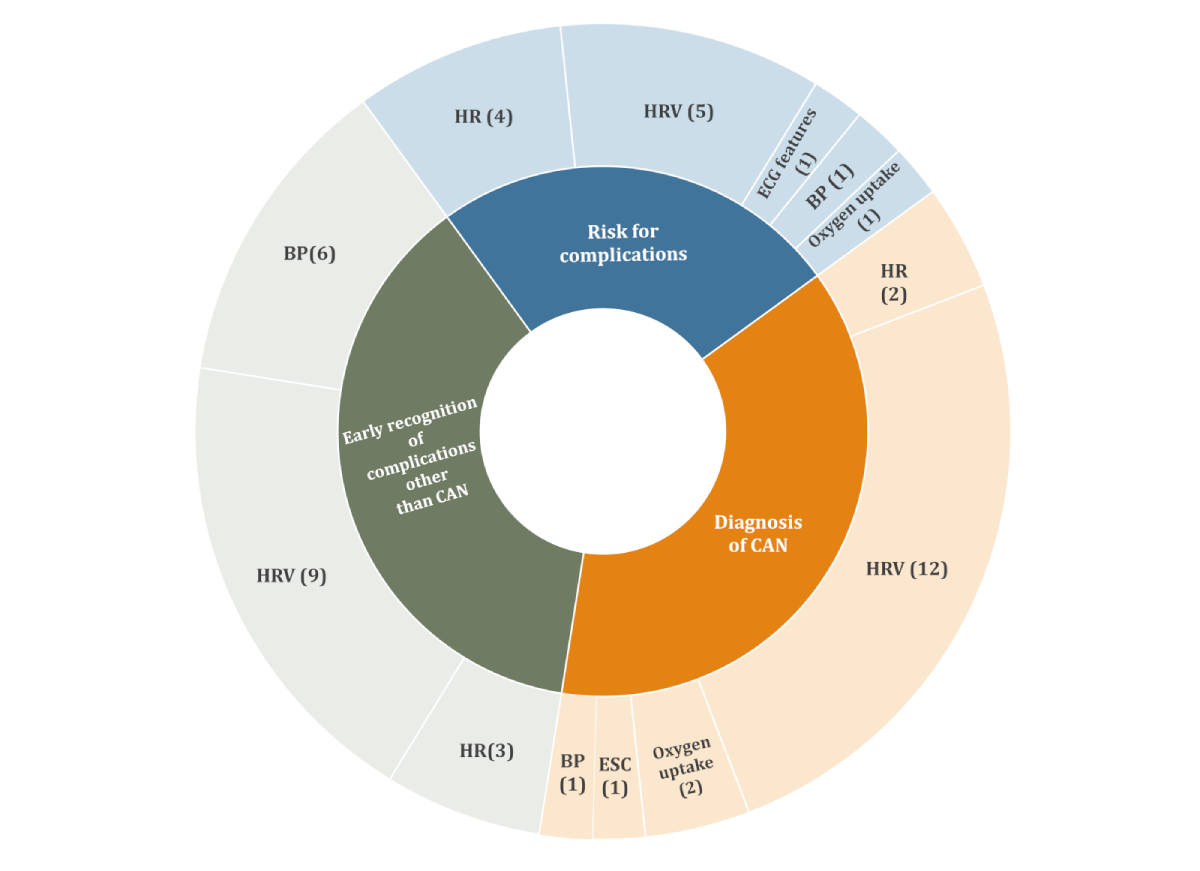
Identified areas of potential impact for wearable devices related to long-term type 1 diabetes complications. The numbers indicate the number of relevant studies found for each physiological signal. BP: blood pressure; CAN: cardiac autonomic neuropathy; ECG: electrocardiogram; ESC: electrochemical skin conductance; HR: heart rate; HRV: heart rate variability.

The potential to improve the life of persons living with T1D using wearable sensors relies on not only the capability to detect differences between persons with T1D and healthy individuals but also on the intra- and interindividual differences within populations with T1D. In this section, we present the studies found in this survey that assessed physiological functions within T1D cohorts and discuss the potential for monitoring these functions with wearables to incorporate into T1D acute glycemic management (Figure 4) and early recognition of long-term complications (Figure 5).

#### Acute T1D Glycemic Management

##### Noninvasive Hypoglycemia Detection

As discussed above, the presence of T1D influences cardiac function and the presence of hypoglycemia exaggerates this with stronger activation of sympathetic activity and inhibition of parasympathetic activity as shown in modified time and frequency indices of HRV [65-67] and QTc prolongation [68-70] (Table S4 in Multimedia Appendix 4). The effect of hypoglycemia on HRV was shown to be the same for people with and without CAN [71]. The signature of hypoglycemia on physiological features was leveraged in 5 studies to investigate methods by which hypoglycemia could be detected noninvasively. Of the 5 studies, 3 (60%) used cardiac features from an ECG (HR and QTc interval) as inputs [72-74] and ML-based techniques for developing relevant models. Elvebakk et al [75] explored noninvasive hypoglycemia detection for persons with and without IAH using cardiac and sudomotor features. The study found that it was only possible to detect hypoglycemia in the group of people without IAH, but it was difficult to reliably detect hypoglycemia in persons with IAH. However, from a second study focused only on persons with IAH, the same group proposed the use of a probabilistic model and the combined use of more cardiac and thermoregulatory features, ECG-derived HR, QTc interval, sudomotor activity, near-infrared and bioimpedance spectroscopy, and achieved a detection *F* score of 88% [76]. Finally, Reddy et al [77], using a wearable sensor and an ML algorithm, showed that the levels of HR and BG before exercise were predictors of exercise-induced hypoglycemia with 80% accuracy.

##### Near-Future Glucose Prediction

Of the 77 studies, 3 (4%) explored the potential of using physiological features monitored with wearable sensors to enhance the accuracy of near-future glucose prediction (Table S5 in Multimedia Appendix 4). The study by Rodriguez et al [78] presented a feature selection method for the estimation of feature importance related to near-future BG prediction and showed that HR and sleep were significant for this task (*P*=.03 and *P*=.04 respectively). This result was confirmed by Hobbs et al [79], who explored the impact on the 30-minute ahead glucose prediction of HR data collected from a commercial wearable device in adults during physical activity. The study showed that HR information led to better model prediction accuracy, assessed over several metrics, such as root mean square error and the Akaike information criterion, and reduced the prediction delay by 2-3 minutes compared with the model that did not consider HR. Mirshekarian et al [80] showed that when ESC and HR were added as features to an ML model for glucose prediction, the performance in terms of root mean square error improved for horizons of 30 and 60 minutes, although the improvement was only small.

##### Glucose Sensing

Of the 77 studies, 3 (4%) explored methods to improve the accuracy of glucose sensing (Table S5 in Multimedia Appendix 4). Here, we identified the work of Laguna et al [81], which aimed to improve the known increase in CGM error during exercise by coupling the CGM with wearable sensors. The study found that when CGMs were coupled with energy expenditure (metabolic equivalent of tasks) and ST information from the wearable sensors, the glucose measurement error dropped from 17.5% to 13.6%. A study by Turksoy et al [82] followed a similar approach whereby the objective was to model the glucose changes during exercise as a function of biometric inputs including HR, GSR, ST, and energy expenditure. The study involved 26 participants who wore wearable sensors for 6 days. The participants performed a series of exercises during the regular clinic visits. The study results showed that ST was the most significant feature describing glucose fluctuation during exercise, followed by HR and energy expenditure. Finally, Rothberg et al [83] explored links between HRV features and absolute BG levels, which were confirmed for persons with type 2 diabetes but not for those with T1D.

##### Integrated Systems Solutions

In view of the broad application range of wearable devices in T1D management, one study identified in this survey focused on the development of an integrated system solution for home-based monitoring and explored the engagement of persons living with T1D using this technology [115].

##### Summary

The aforementioned studies illustrate the potential of using noninvasive biomarkers, which can be monitored with wearable sensors, to improve many aspects in the daily management of T1D (Figure 4). Noninvasive hypoglycemia detection could enhance the recognition of hypoglycemic events in people with IAH and for those who do not use CGMs and rely only on sporadic BG measurements. Near-future glucose prediction is crucial to compensate for the CGM and insulin action delays for more accurate prediction of upcoming hypo- and hyperglycemic events. All the above functionalities are also indispensable for the enhancement of the current AP systems, both for the development of more efficient control algorithms and for the support of the general safety of APs. Although the improvements demonstrated so far have limited clinical significance, these studies are promising first steps. More studies need to be conducted, including a thorough exploration of prediction modeling techniques, as well as the collection of comprehensive data sets that span representative real-life scenarios.

#### Long-term Complications

##### Diagnosis and Monitoring of CAN

As demonstrated by many studies, among the cardiac features, HRV plays a central role in the assessment of autonomic function in general and of CAN in particular [84-88]. Silva et al [89] showed that a high resting HR was associated with reduced parasympathetic activity and lower HRV. Finally, insulin resistance was shown to be associated with lower cardiac output [90] and cardiovascular suppression [91] during and after exercise or cardiac autonomic reflex tests. Anxiety [92] and psychosocial stress [93] were shown to reduce HRV and induce further parasympathetic suppression in persons with T1D, indicating the need to consider confounding factors in assessing CAN. Gender and race were also found to be associated with autonomic function [94]. Finally, a study by Ang et al [95] explored the potential of using ESC as a measure of sudomotor function for the early recognition of CAN but found no significant correlations. However, Riguetto et al [96] showed that postprandial sweating, as well as hypertension, diastolic blood pressure, retinopathy, and nephropathy were independent predictors of CAN. The methods and findings of the above studies are presented in Table S6 in Multimedia Appendix 4.

##### Early Recognition of Complications Other Than CAN

Of the 77 studies, 10 (13%) studies explored other long-term complications of T1D and their relation to cardiovascular autonomic function, including the presence of CAN (Table S7 in Multimedia Appendix 4). In a study by Duvnjak et al [97], the coefficient of variation and spectral indices of HRV correlated with *diabetic retinopathy* development and progression in persons with T1D. In another study of persons with T1D, correlations were demonstrated between HRV indices and neuroretinal layer thickness [98]. A study by Mala et al [99] demonstrated that CAN had a positive association with carotid intima media thickness in T1D and suggested its potential role in the pathogenesis of *atherosclerosis*. HRV is associated with *diabetic kidney disease* as discussed by Sekercioglu et al [100], who showed that, for adults with prolonged T1D duration, the factors of older age at diagnosis and lower HRV might indicate a risk for this complication. CAN in persons with T1D has also been shown to be associated with reduced *bone density* [101], *renal function and albuminuria* [102,103], and *female sexual dysfunction and urinary incontinence* [104]. Finally, in a study by da Silva et al [105], young persons with T1D and increased risk for cardiovascular disease presented greater parasympathetic autonomic dysfunction, whereas a study by Christensen et al [106] suggested that sudomotor function was associated with diabetic *peripheral neuropathy* and could be used as a diagnostic tool for these complications.

##### Risk for Long-term Complications

The quality of glucose control is an uncontroversial marker of the risk of long-term complications. Of the 77 studies, 8 (10%) studies explored the relationship between features of the cardiac and cardiorespiratory function and the quality of glucose control such that their findings could be useful in developing tools for complication risk assessment (Table S8 in Multimedia Appendix 4). Higher levels of HbA_1c_ were shown to correlate with lower HRV levels and higher CAN prevalence [107,108]. A study by Guan et al [109] showed that people with higher HbA_1c_ levels displayed an impaired autonomic response to stress, including a greater change in the HF component of their HRV, whereas Stern et al [110] showed that the QTc interval in persons with T1D correlated with HbA_1c_ and autonomic function. Glucose variability has been shown to correlate with CAN [111] but not with the loss of nocturnal lowering (ie, nondipping) of blood pressure [112]. Similarly, an association between HbA_1c_ and HR dynamics, including the HR-to-performance curve, was demonstrated during cardiopulmonary exercise tests [113], as well as an association between HbA_1c_ and cardiorespiratory fitness as assessed by the time to exhaustion and peak VO_2_ [114]. Finally, the study by Zabeen et al [13] showed that glucose variability, independent of HbA_1c_, plays a role in the development of risk for long-term complications, such as retinopathy and neuropathy.

##### Summary

The evidence presented in the aforementioned studies indicates that autonomic function and CAN could be continuously assessed through wearable devices that provide HRV monitoring. In such a case, the effects of factors such as time of day, meals, exercise, sleep, and glycemic level, as well as therapies for CAN, can be assessed. Moreover, it was shown that autonomic and sudomotor functions could flag risks or onset for a range of T1D long-term complications. Monitoring autonomic function has the potential to complement measures of overall glycemic control and glucose variability in optimizing management to mitigate the risk of long-term complications. Figure 5 summarizes these findings. The relationship found between physiological parameters and quality of glucose control, such as HbA_1c_ or glucose variability, could also be used in the formulation of AP cost functions. In all cases, further studies are needed to investigate how to use the information harvested by wearable sensors toward supporting the early recognition of chronic T1D complications.

## Discussion

### Survey Findings

The data from many studies in this survey showed that a variety of physiological parameters (1) can differentiate persons with T1D from healthy control participants and (2) are associated with aspects of glycemic control, in particular hypoglycemia, and the presence of diabetes complications within cohorts of persons with T1D.

The most explored physiological functions were cardiac autonomic, cardiorespiratory control balance, and thermal homeostasis. The survey identified that monitoring of physiological parameters, such as HR, HRV, QTc, ESC, baroreflex sensitivity, and VO_2_, as well as the autonomic, cardiorespiratory, and thermal homeostasis functions, can be used to identify differences between persons with T1D and healthy control participants. The most pronounced differences between the 2 populations were shown in aspects of cardiac autonomic function, including parasympathetic activity, vagal tone, and cardiac repolarization and depolarization.

With respect to noninvasive hypoglycemia prediction and detection, physiological parameters of the ECG, including HR, HRV, and QTc, have considerable potential to be leveraged. Apart from hypoglycemia detection, these physiological parameters, as well as ESC and ST, can be monitored in conjunction with glucose to compensate for the deficiencies in CGM, such as signal lag, which is vital to the enhancement of AP development.

The screening of long-term T1D complications can also be enhanced. The demonstrated relationship between CAN and HRV paves the way for its continuous, and at-home, assessment. Other complications such as retinopathy and diabetic peripheral neuropathy were shown to relate to cardiac autonomic function and CAN and their onset could be predicted through monitoring of these functions.

Most of the studies discussed in this survey followed conventional statistical analysis methods to assess the existence of correlations in their measured data. ML methods have been used in some studies for the recognition of hypoglycemia and near-future glucose prediction [72-74,77-80].

Most of the surveyed studies used conventional devices; however, the measurement of the above physiological parameters can be performed with the 2020 commercially available wearable, noninvasive sensors. Table 1 lists the identified physiological functions and parameters, together with examples of commercially available wearable sensors that can monitor them today. Only a few studies found in this survey used wearable sensors [64,66,77-82]. These studies mostly focused on the detection of hypoglycemia, glucose prediction, or improvement of CGM glucose measurement accuracy.

The existence of wearable technology that can perform this type of physiological parameter monitoring is a crucial first step in the confirmation of our survey hypothesis that wearables have the potential to enhance T1D sensing with richer information seamlessly and continuously toward improved daily management decisions, and mitigation of complications. In view of this potential, the challenges and perspectives of this endeavor are discussed further below.

**Table 1:** Identified physiological functions and parameters and examples of corresponding commercially available, wearable, noninvasive sensors.

Physiological functions	Physiological parameters	Existing wearable devices
Cardiac autonomic function	HR^a^, HRV^b^ (ECG^c^)	QardioCore and Apple watch
Cardiac repolarization	QT^d^, QTc^e^, T-wave (ECG)	QardioCore and Apple watch
Cardiac output	Bioimpedance	BIOPAC
Energy expenditure	VO_2_^f^	Garmin Forerunner 935, Fitbit Charge 2
Baroreflex sensitivity	ECG, BP^g^	QardioCore, Apple watch
Sweat rate	Sweat rate	KuduSmart monitor
Oxygen saturation	SpO_2_^h^	Withings Pulse Ox; Garmin Fenix 6x
Sudomotor function	ESC^i^	Shimmer3 GSR^j^+ unit
Skin temperature	ST^k^	Tempatilumi CEBrazil; TIDA-00824 Texas Instruments (prototype)

^a^HR: heart rate.

^b^HRV: heart rate variability.

^c^ECG: electrocardiogram.

^d^QT: time interval from the start of the Q-wave to the end of the T-wave in an electrocardiogram.

^e^QTc: QT corrected.

^f^VO_2_: oxygen uptake.

^g^BP: blood pressure.

^h^SpO_2_: oxygen saturation.

^i^ESC: electrochemical skin conductance.

^j^GSR: galvanic skin response.

^k^ST: skin temperature.

### Wearable Sensors Versus Medical Grade Devices

An important parameter when considering the potential of wearables is the quality of the generated data and the ability to extract the required information from them. A clinical ECG setup offers much higher accuracy than a wearable bracelet. Wearable sensors must compromise accuracy for small size, low cost, and high autonomy. Moreover, their default use involves people undertaking daily activities, which introduce motion artifacts and data corruption. Although in most of the studies reported in this survey, the involved participants followed a specified protocol under the supervision of a clinical staff member, this condition cannot be guaranteed or controlled in a daily life setting. To this end, a one-to-one comparison between a medical grade device and its wearable counterpart would always be an uneven battle. However, the claim of wearable sensors is not to substitute medical grade devices but rather to take up the space where the latter cannot be used; that is, the space of at-home, daily life routine. This different use case offers the following critical advantage over medical grade devices: the massive generation of data [24].

Compared with a clinical study, a wearable monitoring scenario produces vastly larger volumes of data over much longer periods and during various conditions, such as sleep, physical activity, eating, resting, stress, and working. Although wearables compromise accuracy, they can offer a significantly better and more representative coverage of a human life’s spectrum. The *big data* return can compensate for data noise by leveraging redundancies and information fusion coming from different wearable sources. Long-term information, such as seasonality, might be revealed, which would not be feasible for short-duration clinical protocols. Finally, the possibility of monitoring a large number of participants opens the potential for population studies that are currently very difficult or very costly to perform. At the same time, efforts for disease management personalization can be significantly boosted by the increase in data availability per user. To this end, the usability of wearable sensors in health care should be considered in light of their space of function, the added value that this space can encompass, and the additional knowledge extraction possibilities that follow from their much larger volume of generated data.

In view of their high data quantity and complexity, wearable devices usually require advanced processing techniques for the harvesting of their data. Although classical signal processing techniques may be sufficient for the extraction of the HRV or QT interval from a medical grade ECG signal, ML strategies may need to be used to perform the same task on ECG data collected from a wearable device. At the same time, there is a requirement for increasingly complex solutions to support clinical judgment and decision-making to meet the current medical and user demands. To this end, wearable-based applications require a postsensing stage of complex data processing to drive usable and viable solutions. The development of processing techniques for information extraction and decision-making based on data generated by wearable sensors is a field that currently receives intensive attention and research. This manifests itself in most of the studies that propose the use of wearables in health care, as discussed in the Introduction and Results sections.

### Impact of Existing Wearable, Noninvasive Sensors on T1D

Despite the depth of research dedicated to the exploration of relations between T1D and other physiological functions, only a few studies found in this survey used wearable sensors. These studies, although still small in number, support the contention that off-the-shelf wearable devices could be readily used in T1D interventions. However, the adoption of this technology in the field is very slow. In addition to the data quality–related challenges, the complex relationships between these biomarkers and glucose regulation provide an additional hurdle. Further research is required for the development of models and simulations, design of treatment strategies for the inclusion of new inputs, and finally, conduction of further clinical trials to demonstrate the added clinical impact of the methods. Finally, the strict safety constraints that need to be guaranteed during the daily management of T1D render the validation of new methods very demanding. In view of these challenges, publicly available data sets have been released to support the research output toward the development of data analytics tools to incorporate information received by wearable devices into T1D interventions [116,117].

In summary, the major challenges in the adoption of wearable technology in the management of T1D and its complications are as follows:

1. Data coming from wearables tend to be noisy, corrupted by motion artifacts, and have lower accuracy than those originating from medical devices.
2. The relationship between the parameters monitored with wearables and glucose regulation is complex.
3. The strict safety constraints in the management of T1D impose hard boundaries on the testing and validation of new decision tools.

### Benefits and Future Directions

Despite the hurdles, this survey makes the case that wearable sensors have a significant potential to enhance the life of those living with T1D. The identified links between physiological parameters that wearables can monitor and T1D can be used to augment the T1D sensing space and develop better management tools. The continuous monitoring potential and the abundance of generated data per person can assist in the personalization of interventions. At the same time, wearable sensors that provide seamless and noninvasive monitoring are expected to add a minimum sensing burden compared with other types of sensors, whereas the automation of management processes that today require the cognitive effort of the persons living with T1D (eg, insulin bolus calculation) or induce stress (eg, fear of hypoglycemia) is expected to lead to better quality of life and lower daily burden.

To harvest this potential, 2 main directions for future research can be identified. First, advanced data processing strategies need to be developed to extract the information obtained from the data collected through wearable sensors. This research direction is not specific to T1D, and T1D can profit from the research outcomes of every field (health care or other), which opts to use wearable sensors. Second, further simulations, models, and clinical studies need to be conducted to support the development of decision tools for T1D based on the data collected through wearables. This direction is T1D-specific and involves multidisciplinary collaboration among data scientists, engineers, clinicians in the field, and persons with lived experience of T1D.

### Comparison With Previous Work

This survey aims to bridge on one side the large volume of research dedicated to identifying correlations between T1D glycemic control and complications with measurable physiological functions and on the other side, the novel potential of wearable technology in medical applications. The bulk of existing work dedicated to wearable technology and T1D is related to glucose sensing and omits other biomarkers that can be readily and noninvasively monitored with the available wearable sensors. To the authors’ knowledge, a survey that explores the potential of wearable sensors in T1D has not been conducted till date. This is the first attempt to bring the fields of T1D and wearables together to highlight the potential of these sensors in the daily management of this disease and the mitigation of its long-term complications.

### Survey Limitations

The main limitation of the survey was that the search was conducted based on a list of wearable-enabled biomarkers (Textbox 1), which may not be exhaustive. Additional knowledge models related to wearables and T1D may exist that were not identified. Moreover, the focus was only on noninvasive sensors and did not consider minimally invasive wearable technology, which can be another pathway to further enhance T1D diagnosis and management. Similarly, the survey focused only on wearable sensors and did not discuss noninvasive, nonwearable devices, such as breath or saliva sensors, which can also be highly advantageous in T1D (eg, breath acetone sensing).

### Conclusions

Considering the wearable sensor boom and its gradual adoption in the health care domain, this survey aimed to investigate their potential impact on T1D, a chronic disease that affects millions of people worldwide and requires daily and costly management and care. The survey search strategy targeted the discovery of studies that examined the relationship between physiological functions or conditions measurable by wearable sensors and T1D. Our analysis showed that T1D greatly affects cardiac, cardiorespiratory, and thermoregulatory functions, and its impact can be readily observed through features of the ECG, such as HRV, QT interval, and T-wave, as well as skin properties such as ESC, temperature, and sweat profile. The effects of T1D on these functions manifest themselves at rest, overnight, during and after exercise, and during daily life activities. Importantly, they can be leveraged to improve the prompt detection of hypoglycemia, the efficiency of the AP, and the diagnosis of CAN and other complications.

Commercially available wearable technology exists for continuous, noninvasive monitoring of the above parameters. For the successful adoption of this technology in health care in general, and T1D in particular, several challenges still need to be resolved, such as issues related to motion artifacts and noise removal, accurate extraction of the features of interest, and development of decision algorithms for improved and safe disease management. Current research efforts are working toward advanced algorithmic solutions for the efficient processing of massive amounts of data produced by wearable sensors. Their promising results can pave the way for similar endeavors for T1D.

## Appendix

Multimedia Appendix 1 Sensing in type 1 diabetes.

Multimedia Appendix 2 Noninvasive wearable sensors in health care.

Multimedia Appendix 3 Survey methodology.

Multimedia Appendix 4 Summary of the studies included in the survey.

## Abbreviations

AP: artificial pancreas
BG: blood glucose
CAN: cardiac autonomic neuropathy
CGM: continuous glucose monitor
ECG: electrocardiogram
ESC: electrochemical skin conductance
GSR: galvanic skin response
HbA_1c_: hemoglobin A_1c_
HF: high frequency
HR: heart rate
HRV: heart rate variability
IAH: impaired awareness to hypoglycemia
LF: low frequency
ML: machine learning
PRISMA: Preferred Reporting Items for Systematic Reviews and Meta-Analyses
QTc: QT corrected
SpO_2_: oxygen saturation
ST: skin temperature
T1D: type 1 diabetes
VO_2_: peak oxygen uptake
