# Supplementary material for: The Potential of Current Noninvasive Wearable Technology for the Monitoring of Physiological Signals in the Management of Type 1 Diabetes: Literature Survey
Source: J Med Internet Res. 2022 Apr 8;24(4):e28901. doi: 10.2196/28901 (PMC9034434; doi:10.2196/28901)
Supplement: Multimedia Appendix 1 [file jmir_v24i4e28901_app1.docx]

# Multimedia Appendix 1

## Sensing in Type 1 Diabetes.

To date, conventional type 1 diabetes (T1D) management relies almost solely on glucose monitoring to guide therapeutic decisions. Glucose measurements are usually taken using capillary blood obtained by a finger prick using a blood glucose meter and increasingly on interstitial glucose measurements made by continuous glucose monitors (CGM). Finger prick capillary blood measurements are invasive and painful and can only provide measurements at a single point in time without any indication of glucose trends. CGM offers continuous and minimally invasive sensing however the measurements are is less accurate than glucose meters and are associated with sensing lags (in the order of 10 minutes) [1], [2]. Blood ketone monitoring by finger pricking has been introduced over the last decade in the daily management of T1D in order to assess the onset of diabetic ketoacidosis, a common severe complication arising from very high glucose levels.

For long-term complications, assessment of cardiac autonomic neuropathy (CAN) is usually based on electrocardiogram (ECG) monitoring while the patient is at rest or performs cardiac autonomic reflex tests [3], [4]. Other diagnostic methods exist such as the baroreceptor reflex and the heart rate turbulence method. Diabetic retinopathy is diagnosed through a dilated eye optometry exam, while nephropathy is diagnosed by albumin detection in urine supported potentially by x-rays and ultrasound. Other T1D long-term complications require different diagnostic procedures based on various associated biomarkers. Conclusively, a wide range of monitoring methods, diverse in the type of data, as well as in their temporal and spatial characteristics, are involved in the diagnosis of T1D long-term complications.

### Challenges in T1D sensing

Reliance upon monitoring of glucose alone, even when performed continuously, does not guarantee a metabolic stability and may not prevent hypoglycemia. Numerous situations where insulin requirements may change rapidly such as with meal consumption or exercise may not be addressed in a timely manner as a consequence of the delays in insulin action and interstitial glucose sensing. These limitations call for proactive strategies to anticipate insulin needs under these circumstances. Moreover, the metabolic heterogeneity of those living with T1D raises the need for deep and real-time personalization in insulin dosing responses [5] which cannot be adequately addressed by glucose monitoring alone. These issues represent significant challenges to the development of an artificial pancreas with fully closed-loop functionality.

Another major issue relates with the timeliness and effectiveness in the early recognition of long-term complications. In all cases, diagnosis requires specialized equipment and personnel and is performed at a clinical environment. This limits accessibility and results in delays to diagnosis. Moreover, the diagnosis is bounded by the information collected during the time of the visit which is often not reflective of the overall state of the individual or their state during daily life activities. As an example, in the case of CAN, a standard diagnostic procedure is currently missing [6] and the above issues are reflected in the very high variance of prevalence statistics reported by different studies [7].

The above issues indicate the need to augment the T1D sensing space with additional biomarkers in order to establish a more complete picture of the current status and trends of the disease, which will assist in better acute management and prompt recognition of long-term complications. But, while this endeavor is arising from the clinical facts, it is important to also consider the psychological and emotional aspects of managing the disease. People living with T1D struggle with the number of devices and supplies they must constantly carry with them, and be responsible for, and report challenges related to the cost, visibility and intrusiveness of the devices [8]. These issues signal the difficulty of balancing the need for additional monitoring with the emotional well-being of people with T1D.

To date, non-invasive wearable sensing devices are used daily by millions of people worldwide. Although initially designed for physical activity and wellness monitoring, wearable sensors are now turning towards physiological signal monitoring and health management. This fact renders wearables an excellent candidate to bridge the gap between the addition of biomarkers and the user adoption trade-off in T1D. In view of their rapidly increasing acceptance and market penetration, this survey aims to investigate the existence of T1D biomarkers that can be measured with the currently available wearable, non-invasive technology and explore the challenges and perspectives of such an endeavor towards enhancing T1D interventions.

## References

1. Schmelzeisen-Redeker G, Schoemaker M, Kirchsteiger H, Freckmann G, Heinemann L, del Re L. Time Delay of CGM Sensors. J Diabetes Sci Technol. 2015 Aug 4;9(5):1006–1015. PMID:26243773

2. Zaharieva DP, Turksoy K, McGaugh SM, Pooni R, Vienneau T, Ly T, Riddell MC. Lag Time Remains with Newer Real-Time Continuous Glucose Monitoring Technology During Aerobic Exercise in Adults Living with Type 1 Diabetes. Diabetes Technol Ther. 2019;21(6):313–321. PMID:31059282

3. Serhiyenko VA, Serhiyenko AA. Cardiac autonomic neuropathy: Risk factors, diagnosis and treatment. World J Diabetes. 2018 Jan 15;9(1):1–24. PMID:29359025

4. Pop-Busui R. Cardiac Autonomic Neuropathy in Diabetes: A clinical perspective. Diabetes Care. 2010 Feb 1;33(2):434–441. PMID:20103559

5. Donsa K, Spat S, Beck P, Pieber TR, Holzinger A. Towards Personalization of Diabetes Therapy Using Computerized Decision Support and Machine Learning: Some Open Problems and Challenges. In: Holzinger A, Röcker C, Ziefle M, editors. Smart Health Open Probl Future Chall. 2015:237–260. Springer International Publishing. doi: 10.1007/978-3-319-16226-3_10

6. Bissinger A. Cardiac Autonomic Neuropathy: Why Should Cardiologists Care about That? [Internet]. J Diabetes Res. Hindawi; 2017:e5374176. doi: 10.1155/2017/5374176

7. Vinik AI, Maser RE, Mitchell BD, Freeman R. Diabetic Autonomic Neuropathy. Diabetes Care. 2003 May 1;26(5):1553–1579. PMID:12716821

8. Brew-Sam N, Chhabra M, Parkinson A, Hannan K, Brown E, Pedley L, Brown K, Wright K, Pedley E, Nolan CJ, Phillips C, Suominen H, Tricoli A, Desborough J. Experiences of Young People and Their Caregivers of Using Technology to Manage Type 1 Diabetes Mellitus: Systematic Literature Review and Narrative Synthesis. JMIR Diabetes. 2021 Feb 2;6(1):e20973. doi: 10.2196/20973
