# Supplementary material for: The Potential of Current Noninvasive Wearable Technology for the Monitoring of Physiological Signals in the Management of Type 1 Diabetes: Literature Survey
Source: J Med Internet Res. 2022 Apr 8;24(4):e28901. doi: 10.2196/28901 (PMC9034434; doi:10.2196/28901)
Supplement: Multimedia Appendix 2 [file jmir_v24i4e28901_app2.docx]

# Multimedia Appendix 2

## Noninvasive wearable sensors in healthcare

### Wearable sensors

According to Gao et al. [1], wearable sensors (a.k.a. wearable devices or wearables) are *“devices that can be worn or mated with human skin to continuously and closely monitor an individual's activities, without interrupting or limiting the user's motions''*. Wearable sensors can be classified based on their invasiveness as minimally invasive, requiring a small insertion into the body, and non-invasive, which do not introduce any tissue disruption. An example of a minimally invasive wearable sensor is the CGM, and of a non-invasive one is the heart rate (HR) monitor. In this study we focus only on non-invasive wearable sensors. Wearable sensors present the advantage of, continuous and seamless monitoring which, combined with their low cost, leads to their increasing acceptance and adoption from a large portion of the population. Boosted by their smaller size, better functionality, higher autonomy, and improved accuracy, wearables are becoming more and more integrated in our everyday life with a vast number and type of products available in the market today; indicatively, the value of the wearables market was $1.1 billion in 2019 and is expected to grow to $8.6 billion by 2024 [2]. Non-invasive, wearable sensors are usually worn as belts, arm bands, smart textiles [3], smart glasses, earphones or bracelets, the latter including the broad category of smart watches (see Fig. 1 of main paper).

Although originally dedicated to activity tracking through accelerometers and gyroscopes [4], to date, wearable sensors enable the continuous, non-invasive monitoring of various physiological signals, including HR, breath rate, oxygen saturation, skin temperature, and electrochemical skin conductance or galvanic skin response. Recently, belt and bracelet-type wearable devices (e.g. QardioCore and Apple Watch) can provide ECG monitoring, while a vast research and market effort is on board for the enhancement of wearables with non-invasive, continuous BP monitoring [5]. The introduction of physiological parameters monitoring, and the improvement of the sensing accuracy is presently steering the application space of wearable sensors towards the domain of healthcare [6]. These devices can enable continuous health monitoring for early detection of dangerous health conditions and at-home disease management [7]. At the same time, they bring a significant socio-economic impact by enabling remote and low-cost health management services, which would be highly beneficial for people living in rural or poor areas, with lower accessibility to medical setups [8].

### Wearable sensors in healthcare

The development of healthcare applications based on data generated by wearable devices is a field that currently receives intensive attention and research. Usually, these applications combine wearables with advanced data processing techniques, such as machine learning in order to manage the size of the produced data, mitigate the motion artefacts and generate smart decisions. Examples include the work of Airaksinen et al. [9] in which wearable accelerometers and gyroscopes combined with Convolutional Neural Networks were used to record the motion of infants with the aim to detect risk for neurodevelopmental disorders. In the study be Omer et al. [10] heart failure patients under moderate intensity exercise were equipped with a wearable ECG and vibration–based cardiogram sensing patch and a ML method graphical technique was developed in order to assess their clinical status and treatment response. Finally, in the study of Yin et al. [11] wearable sensors were used for the diagnosis of different medical conditions such as arrhythmia and hypothyroid based on a comparative assessment of a large set of ML algorithms. These promising results demonstrate the broad potential of wearable sensors in healthcare and at the same time highlight the need for advanced algorithmic solutions for the efficient processing of their data. Many challenges are still there; novel processing and interpretation frameworks, new clinical procedures, data security and ethics are yet to be addressed prior to adoption of this technology in practical healthcare settings [12].

Despite the progress in other healthcare domains, for the case of T1D, although there exist studies on the use of wearables for the detection of physical activity [13], [14], direct applications of wearables in the acute management and/or the diagnosis of long-term complications of the disease are very limited, as we will see in this survey. In order to boost the research and development of novel T1D applications based on wearable devices, it is important to investigate systematically if and how the information monitored by those sensors is related to aspects of T1D management and diagnosis of complications.

## References

1. Gao W, Emaminejad S, Nyein HYY, Challa S, Chen K, Peck A, Fahad HM, Ota H, Shiraki H, Kiriya D, Lien D-H, Brooks GA, Davis RW, Javey A. Fully integrated wearable sensor arrays for multiplexed *in situ* perspiration analysis. Nature. 2016 Jan;529(7587):509–514. doi: 10.1038/nature16521

2. Research and Markets. Industrial Wearables Market by Device Type (AR Glasses, VR Headsets, Smartwatches, Smart Bands), Industry (Automotive, Aerospace, Manufacturing, Oil & Gas, Power & Energy), Component, and Region - Global Forecast to 2024. https://www.researchandmarkets.com/reports/4868114/industrial-wearables-market-by-device-type-ar?utm_source=dynamic&utm_medium=BW&utm_code=2jrxzg&utm_campaign=1329406+-+Global+Industrial+Wearables+Market+size+is+Projected+to+grow+from+USD+1.1+Billion+in+2019+to+USD+8.6+Billion+by+2024&utm_exec=anwr281bwd

3. Fernández-Caramés TM, Fraga-Lamas P. Towards the Internet of Smart Clothing: A Review on IoT Wearables and Garments for Creating Intelligent Connected E-Textiles. Electronics Multidisciplinary Digital Publishing Institute; 2018 Dec;7(12):405. doi: 10.3390/electronics7120405

4. Mehrang S, Pietilä J, Korhonen I. An Activity Recognition Framework Deploying the Random Forest Classifier and A Single Optical Heart Rate Monitoring and Triaxial Accelerometer Wrist-Band. Sensors Multidisciplinary Digital Publishing Institute; 2018 Feb;18(2):613. doi: 10.3390/s18020613

5. Rachim VP, Chung W-Y. Multimodal Wrist Biosensor for Wearable Cuff-less Blood Pressure Monitoring System. Sci Rep. 2019 May 28;9(1):1–9. doi: 10.1038/s41598-019-44348-3

6. Witt D, Kellogg R, Snyder M, Dunn J. Windows Into Human Health Through Wearables Data Analytics. Curr Opin Biomed Eng. 2019 Mar;9:28–46. PMID:31832566

7. Haghi M, Thurow K, Stoll R. Wearable Devices in Medical Internet of Things: Scientific Research and Commercially Available Devices. Healthc Inform Res. 2017 Jan 1;23(1):4–15. doi: 10.4258/hir.2017.23.1.4

8. Song Y, Tan Y, Song Y, Wu P, Cheng JCP, Kim MJ, Wang X. Spatial and temporal variations of spatial population accessibility to public hospitals: a case study of rural–urban comparison. GIScience Remote Sens. 2018 Sep 3;55(5):718–744. doi: 10.1080/15481603.2018.1446713

9. Airaksinen M, Räsänen O, Ilén E, Häyrinen T, Kivi A, Marchi V, Gallen A, Blom S, Varhe A, Kaartinen N, Haataja L, Vanhatalo S. Automatic Posture and Movement Tracking of Infants with Wearable Movement Sensors. Sci Rep. 2020 Jan 13;10(1):1–13. doi: 10.1038/s41598-019-56862-5

10. Inan Omer T., Baran Pouyan Maziyar, Javaid Abdul Q., Dowling Sean, Etemadi Mozziyar, Dorier Alexis, Heller J. Alex, Bicen A. Ozan, Roy Shuvo, De Marco Teresa, Klein Liviu. Novel Wearable Seismocardiography and Machine Learning Algorithms Can Assess Clinical Status of Heart Failure Patients. Circ Heart Fail. 2018 Jan 1;11(1):e004313. doi: 10.1161/CIRCHEARTFAILURE.117.004313

11. Yin H, Jha NK. A Health Decision Support System for Disease Diagnosis Based on Wearable Medical Sensors and Machine Learning Ensembles. IEEE Trans Multi-Scale Comput Syst. 2017 Oct;3(4):228–241. doi: 10.1109/TMSCS.2017.2710194

12. Izmailova ES, Wagner JA, Perakslis ED. Wearable Devices in Clinical Trials: Hype and Hypothesis. Clin Pharmacol Ther. 2018;104(1):42–52. doi: 10.1002/cpt.966

13. Yavelberg L, Zaharieva D, Cinar A, Riddell MC, Jamnik V. A Pilot Study Validating Select Research-Grade and Consumer-Based Wearables Throughout a Range of Dynamic Exercise Intensities in Persons With and Without Type 1 Diabetes: A Novel Approach. J Diabetes Sci Technol. 2018 May 1;12(3):569–576. doi: 10.1177/1932296817750401

14. Castle JR, Youssef JE, Wilson LM, Reddy R, Resalat N, Branigan D, Ramsey K, Leitschuh J, Rajhbeharrysingh U, Senf B, Sugerman SM, Gabo V, Jacobs PG. Randomized Outpatient Trial of Single- and Dual-Hormone Closed-Loop Systems That Adapt to Exercise Using Wearable Sensors. Diabetes Care. 2018 Jul 1;41(7):1471–1477. PMID:29752345
