# Supplementary material for: The Potential of Current Noninvasive Wearable Technology for the Monitoring of Physiological Signals in the Management of Type 1 Diabetes: Literature Survey
Source: J Med Internet Res. 2022 Apr 8;24(4):e28901. doi: 10.2196/28901 (PMC9034434; doi:10.2196/28901)
Supplement: Multimedia Appendix 3 [file jmir_v24i4e28901_app3.docx]

# Multimedia Appendix 3

## Survey methodology

The search was conducted according to the PRISMA guidelines [1]. Two databases were interrogated: i) PubMed with a timeframe from 01-January-2016 to 01-January-2021, and ii) Scopus with a timeframe from 2016 to 2020. These databases were chosen as they are the principal databases where medical and biomedical engineering studies are included.

Our search query was formed by combining keywords related to T1D with the physiological parameters that can be monitored with wearable, non-invasive sensors available in 2020 as: Diabetes AND ("Type 1" OR "Type one" OR juvenile) AND (“heart rate” OR “heart rate variability” OR “respiration rate” OR “respiratory rate” OR “breath rate” OR “breathing rate” OR “respiration variability” OR “galvanic skin response” OR “skin conductance” OR “skin impedance” OR sweat OR accelerometer* OR gyroscope* OR "oxygen saturation" OR SpO2).

A set of exclusion criteria was defined in order to screen the resulting articles and select the ones relevant to our review research question:

1. Studies that did not involve human subjects or human data.
2. Studies related to drug testing, drug discovery, or chemical compounds.
3. Studies not referring to T1D.
4. Studies based on clinical parameters that cannot be monitored with existing wearable technology (e.g. retina SpO2, arterial stiffness).
5. Studies that discussed wearables for exercise or activity detection as this was considered an indirect link to T1D.
6. Review papers, study protocols, qualitative (case) studies or studies that did not use quantitative methods or did not produce statistically assessed outcomes (e.g. one case reports)

The article selection process was performed in four steps (see Fig. 2 of main paper) following the PRISMA guidelines:

1. **Identification phase**: The search query was applied in PubMed and Scopus with the time constraints discussed above. 626 articles were identified in total.
2. **Screening phase**: From the 626 articles, 226 were excluded in the screening phase; 168 referring to non-human studies, 53 review papers, study protocols and non-quantitative studies, and 3 duplicates. Two additional articles were excluded because the abstract was not available. 400 articles were considered for the next phase.
3. **Eligibility phase**: At this stage, the 400 screened articles were assessed and 323 were excluded because they met one or more of the exclusion criteria. Specifically, 76 articles for being unrelated to T1D, 49 for discussing exercise monitoring with wearables, 75 related to drug or chemical studies, and 123 for studying physiological parameters that cannot be monitored with existing wearable devices. At the end of this phase 77 articles were selected for review.
4. **Inclusion phase**: The inclusion phase comprised the process of reviewing the 89 eligible articles retained at the previous stage.

The eligibility phase of the review process was performed by three independent reviewers with research backgrounds in engineering, sensing, machine learning, signal processing, human-computer interaction, and diabetes. The two reviewers decided independently on the inclusion/exclusion of the query-returned articles based on the above-defined criteria. The third reviewer provided input on those articles where the two other reviewers did not agree. The process was concluded by discussion among the three reviewers and convergence on a unanimous decision.

## References

1. Liberati A, Altman DG, Tetzlaff J, Mulrow C, Gøtzsche PC, Ioannidis JPA, Clarke M, Devereaux PJ, Kleijnen J, Moher D. The PRISMA Statement for Reporting Systematic Reviews and Meta-Analyses of Studies That Evaluate Health Care Interventions: Explanation and Elaboration. PLoS Med. 2009 Jul 21;6(7):e1000100. doi: 10.1371/journal.pmed.1000100
