# Supplementary material for: The Potential of Current Noninvasive Wearable Technology for the Monitoring of Physiological Signals in the Management of Type 1 Diabetes: Literature Survey
Source: J Med Internet Res. 2022 Apr 8;24(4):e28901. doi: 10.2196/28901 (PMC9034434; doi:10.2196/28901)
Supplement: Multimedia Appendix 4 [file jmir_v24i4e28901_app4.docx]

# Multimedia Appendix 4

## Summary of the studies included in the survey

Table S1: Summary of the articles that explored the impact of T1D on the cardiovascular autonomic function

| **Article** | **Aim** | **State** | **Cohort** | **Phys. param** | **Results** |
| --- | --- | --- | --- | --- | --- |
| Wilson et al. (2017) [20] | Association of HRV with exercise capacity in T1D | Rest  CART | 23 T1D  17 controls (C) | HR,  HRV (frequency) | Resting HR elevated and resting HRV reduced in T1D; this was undetected by the CARTs |
| Metwalley et al. (2018) [21] (abstract only available) | Cardiac autonomic function in T1D | Rest  CART | 60 T1D  N/A C  (children) | HR | Parasympathetic/sympathetic autonomic dysfunctions in T1D |
| da Silva et al. (2017) [22] | Compare HRV indices in T1D | Rest | 39 T1D  43 C  (young) | HRV (time, frequency) | Decrease in sympathetic and parasympathetic activity in T1D |
| Ribeiro et al. (2017) [23] | HRV in community-dwelling elders with T1D | Rest | 61 T1D  168 C  (elders) | HRV (time, frequency) | Reduction in HRV time domain indices, not in frequency, in T1D |
| Giacon et. al (2016a) [24] | Autonomic modulation induced by orthostatic test in children with T1D | Orthostatic test | 16 T1D  19 C  (children) | HR, HRV | Children with T1D presented reduced overall variability and parasympathetic modulation |
| Souza et al. (2016) [25] | Nonlinear and linear indices of HRV in T1D | Rest | 20 T1D  23 C | HRV (linear, non-linear) | Autonomic nervous system behavior that tends to randomness in T1D |
| de Oliveira et al. (2018) [26] | HRV symbolic analysis and Shannon entropy in T1D | Rest | 39 T1D  43 C | HRV (linear, symbolic) | Modified linear indexes and symbolic analysis, but not yet complexity of HRV in T1D |
| Garner et al. (2018) [27] | Higuchi and Katz fractal dimension of HRV in T1D | Rest | 23 T1D  23 C | HRV (Katz’s fractal dimension) | Significantly reduced chaotic response, as measured by Katz’s fractal dimension, of HRV in T1D |
| Giacon et al (2016b) [28] | Autonomic modulation in T1D | Rest | 13 T1D  23 C | HRV (geometric indices) | Reduced HRV and parasympathetic modulation in T1D |
| Leite et. al (2019) [29] | Effects of sedentary lifestyle in HRV in T1D | Exercise | 8 T1D  8 C | HRV (time, frequency) | Reduction in HRV time and frequency domain during exercise in T1D |
| Macartney et al. (2020) [30] | HRV during exercise-heat stress in T1D | Rest  Exercise  Recovery | 14 T1D  14 C | HRV (entropy), Body temp. | Body temperature increased more, HRV attenuated more during vigorous exercise in T1D compared to controls |
| Brock et al. (2017) [31] | Relation of cardiac vagal tone with T1D | Rest | 65 T1D  65 C | Vagal tone, HRV (time, frequency) | Cardiac vagal tone in T1D with established neuropathy lower and positively associated with HRV time and frequency |
| Isaksen et al. (2018) [[32]](https://pubmed.ncbi.nlm.nih.gov/29797352/) | Cardiac de-/repolarization durations in T1D | Rest | 855 T1D 1710 C | ECG features | Cardiac depolarization increased at all ages, whereas repolarization increased only in young people with T1D |
| Inanir et al. (2020) [33] | ECG markers of arrhythmias in T1D | Rest | 46 T1D  46 C | HR,  ECG features | HR, QTc and other ECG features significantly higher in T1D |
| Isaksen et al. (2018) [34] | Cardiac repolarization in T1D | Rest | 855 T1D 1710 C | ECG features | T-waves flatter and more asymmetric in T1D |

Table S2: Summary of the articles that explored the impact of T1D on the cardio-respiratory balance and fitness

| **Article** | **Aim** | **State** | **Cohort** | **Phys. parameters** | **Results** |
| --- | --- | --- | --- | --- | --- |
| Moser et al. (2018) [35] | HR during cardio-pulmonary exercise in individuals with T1D compared controls | Exercise | 14 T1D  7 C | HR, VO_2_ | Clear differences in the HR response during cardio-pulmonary exercise |
| Bianchi et al. (2017) [36] | Reciprocal interaction of cardiovascular and respiratory control mechanisms in T1D | Rest | 46 T1D 103 C | ECG, SpO_2_,  BS | Resting SpO2 and BS reduced in T1D. A reduced sensitivity to hypoxia seems a primary factor to reflex sympathetic activation |
| Turinese et al. (2017) [37] | Exercise capacity in people with T1D | Exercise | 17 T1D 17 C | SpO_2_, VO_2_, RER | In T1D worse exercise tolerance |
| Röhling et al. (2017) [38] | Cardio-respiratory function and HRV in T1D | Clamp, Exercise | 163 T1D 40 C | VO_2_, HRV (time, frequency) | Cardiac autonomic function reduced and strongly associated with cardio-respiratory function in T1D |
| Mourot et al. (2020) [39] | Cardiovascular response to exercise in T1D (trained) | Rest  Recovery | 9 T1D  9 C | HR, BS | Similar between T1D trained and controls |
| Francescato et al. (2020) [40] | Glucose pulse relation to T1D (trained) | Exercise | 7 T1D  7 C | HR | Similar between T1D trained and controls |
| Nascimento et al. (2017) [41] | Fitness in non-sedentary people with T1D | Exercise | 21 T1D  16 C | HR, VO_2_ | Similar between T1D trained and controls |
| Ansell et al. (2020) [42] | Short-term effects of high-intensity aerobic exercise on cardiovascular variables in normal weight adolescents with T1D | Exercise | 15 T1D 28 C  adolesc. | Energy expenditure, HRV | Normal weight adolescents with T1D have impaired autonomic function and increased energy expenditure compared to controls. Acute cardiometabolic responses to exercise are normal in T1D with adequate glycemic control. |

Table S3: Summary of the articles that explored the impact of T1D on the thermal homeostasis

| **Article** | **Aim** | **State** | **Cohort** | **Phys. parameters** | **Results** |
| --- | --- | --- | --- | --- | --- |
| Gandecka et al. (2017) [43] | Sudomotor function in adults with T1D | Rest | 404 T1D, 84 C | ESC | Lower ESC in T1D |
| Fujii et al. (2016) [44] | Sweating modulation in T1D | Exercise | 11 T1D 12 C | sweat rate | In individuals with T1D performing moderate intensity exercise in the heat, sweating is modified compared to controls |
| Barone et al. (2018) [45] | Peripheral temperature rhythmicity and control in T1D | Rest | 12 T1D  8 C | body temp. | Similar maximum and higher minimum peripheral body temperature between T1D and controls |

Table S4: Summary of the articles that assessed physiological functions on the non-invasive hypoglycemia detection

| **Article** | **Aim** | **State** | **Cohort** | **Phys. parameters** | **Results** |
| --- | --- | --- | --- | --- | --- |
| Silva et al. (2017) [46] | HRV during hypoglycemia | CART | 99 T1D | HRV (frequency) | Moderate/severe hypoglycemia led to lower HRV (HF) |
| Bekkink et al. (2019) [47] | HRV during hypoglycemia | Daily activities | 23 T1D adults | HRV  (time, frequency) | Hypoglycemia causes early changes in HRV detectable by a wearable device |
| Pertseva et al. (2018) [48] | HRV in relation to T1D duration and glycemic control | Daily activities | 43 T1D adults | HRV (time, frequency) | Hypoglycemic conditions lead to even greater reduction of HRV |
| Koivikko et al. (2017) [49] | Repolarization during nocturnal hypoglycemia | Overnight | 11 T1D adults | ECG features | Spontaneous nocturnal hypoglycemia results in morphological changes and increased heterogeneity of global cardiac repolarization |
| Novodvorsky et al. (2017) [50] | Cardiac arrhythmias, HRV, repolarization during hypoglycemia | Daily activities | 37 T1D <50 years | Arrythmias  HRV (time, frequency), ECG features | Prolonged QTc, T-peak, T-end interval, decreased T-wave symmetry during hypoglycemia |
| Alimova et al. (2018) [51] | Heart rhythm in children with T1D depending on the night level of glycemia | Daily activities | 50 T1D children | HR, QTc | Night hypo- and hyperglycemia in children and adolescents with T1D leads to an extension of the QTc interval |
| Cichosz et al. (2017) [52] | Changes in HRV during hypoglycemia confounded by CAN | CART | 21 T1D adults | HRV (frequency) | Measurable changes in HRV during hypoglycemia with and without CAN |
| Ling et al. (2016) [53] | Non-invasive hypoglycemia monitoring | Rest, Overnight | 16 T1D children | HR, QTc | Sensitivity 78.00%, specificity 60.00% |
| San et al. (2016) [54] | Non-invasive hypoglycemia monitoring | Rest, Overnight | 15 T1D children | HR, QTc | Sensitivity 80%, specificity 50% |
| Ling et al. (2017) [55] | Non-invasive hypoglycemia monitoring | Rest, Overnight | 15 T1D children | HR, QTc | Sensitivity 79.07%, specificity 53.64 % |
| Elvebakk et al. (2018) [56] | Non-invasive hypoglycemia monitoring with IAH | Clamp | 20 T1D adults with IAH | HR  Sweating,  ST,  ECG features | Insufficient sensitivity and specificity |
| Elvebakk et al. (2019) [57] | Non-invasive hypoglycemia monitoring with IAH | Clamp | 20 T1D adults with IAH | HR,  Sudomotor function,  ECG features, Near-infrared, Bioimpedance | Sensitivity 95%, specificity 96%, F score 88%; Important features: QT, wrist and abdomen sudomotor activity |
| Reddy et al. (2019) [58] | Hypoglycemia prediction at the start of exercise | Exercise | 43+12 T1D adults | HR,  Metabolic energy expenditure | Accuracy 79.55%; Two critical features predictive of hypoglycemia during exercise: HR and glucose at the start of exercise |

Table S5: Summary of the articles that assessed physiological functions on the near future glucose prediction and glucose sensing

| **Article** | **Aim** | **State** | **Cohort** | **Phys. parameters** | **Results** |
| --- | --- | --- | --- | --- | --- |
| Rodríguez-Rodríguez et al. (2019) [59] | Feature selection and ranking for glucose prediction | Daily life | 25 T1D | HR,  no of steps,  sleep | Results indicate that the most remarkable variables are insulin, meal, and past glycemia followed by exercise and then HR, sleeping time, and schedule; Some changes of heartbeat are not due to any cause that alters glycemia |
| Hobbs et al. (2019) [60] | Glucose prediction during exercise | Exercise (ski) | 35 T1D adolescents | HR | The addition of HR yields a statistically significant improvement in RMSE |
| Mirshekarian et al. (2019) [61] | Glucose prediction | Daily life | 6 T1D | HR, ST, ESC | When ESC and HR are added they improve the results for both 30 and 60 minutes |
| Laguna et al. (2019) [62] | Enhancement of CGM accuracy during exercise | Exercise | 6 T1D | HR, Metabolic Equivalent of Tasks, ST, GSR | Reduction of CGM error during physical activity (17.46% vs. 13.8%, p < 0.005) to the magnitude of the baseline error level (13.61%); Identified optimal signals are Metabolic Equivalent of Tasks and ST |
| Turksoy et al. (2017) [63] | Correlation between biometric variables and glucose during exercise | Exercise | 26 T1D adults | HR, ST, body temperature, GSR, energy expenditure, 2D acceleration | Different signals are important for different types of exercise; ST is found to be the most consistent important variable that can describe glucose changes during and after exercise |
| Rothberg et al. (2016) [64] | Relationship between BG and HRV | Rest, Postprandial | 32 T1D adults | HRV (frequency) | LF, HF, and total power of HRV negatively associated with BG in T1D. LF/HF positively correlated with BG |

Table S6: Summary of the articles that assessed physiological functions on the detection of CAN

| **Article** | **Aim** | **State** | **Cohort** | **Phys. parameters** | **Results** |
| --- | --- | --- | --- | --- | --- |
| Carricarte Naranjo et al. (2017) [66] | Permutation entropy of HRV for the assessment of CAN | CART | 18 T1D  18 C  (adults) | HRV (time, frequency, entropy) | Permutation entropy of HRV is promising for the clinical quantitative evaluation of CAN |
| Razanskaite-Virbickiene et al. (2017) [67] | HRV (time and frequency) for detecting CAN in youngs with T1D | CART | 67 T1D (15-25 years old) | HRV (time, frequency) | HRV (time and frequency) informative; the HRV coefficient of variation during deep breathing could be valuable for detecting CAN |
| Pan et al. (2019) [68] | Prevalence of CAN among sampled Chinese people with T1D | Ewing’s test | 73 T1D | HR, HRV (time) | Combination of Valsalva and lying-to-standing test was found to be optimal for CAN diagnosis in T1D |
| Carricarte Naranjo et al. (2018) [69] | Comparison of Rényi and permutation entropy for CAN identification in T1D | N/A | 32 T1D (18 without, 14 with CAN) | HRV (entropy indices) | Rényi entropy and permutation entropy provide complementary information achieving 100% classification accuracy in CAN detection |
| Kane et al. (2020) [70] | Non-linear complexity of HRV for CAN identification | Rest | 19 T1D  17 C | HRV (linear, non-linear) | Differences in HRV frequency domain and entropy but not in time-domain |
| Silva et al. (2017) [71] | Associations between HRV and physical activity, body composition, and metabolic and cardiovascular parameters | Rest | 39 T1D | HR, RR, HRV (time, frequency) | High values of at-rest HR were associated with reduced parasympathetic activity and global HRV |
| Niedzwiecki et al. (2017) [72] | Hemodynamic parameters during maximal exercise in men with T1D differing in insulin resistance | Exercise | 40 T1D men | VO_2_ | The higher the insulin resistance the lower the cardiac output during maximal exercise in men with T1D |
| Ziegler et al. (2018) [73] | Association of HRV with insulin resistance and insulin secretion in recent-onset T1D | Clamp,  CART | 275 t1d | HRV (time, frequency) | Insulin resistance may contribute to the development of early cardiovagal suppression rather than sympathetic predominance |
| Bashir et al. (2018) [74] | Correlation between anxiety levels and HRV in adolescents with T1D | Rest | 33 T1D  31 C (adol.) | HRV  anxiety | Anxiety appears to play an important role in reducing HRV in patients with T1D |
| Kristiansen et al. (2020) [75] | Impact of psychosocial stress on HRV in T1D | Rest | 113 T1D  54 C | HRV, psychosocial stress | Psychosocial stress might be a confounder for reduced HRV |
| Faulkner et al. (2019) [76] | Association of sex and race with HRV and cardiorespiratory fitness in adolescents with T1D | Rest, Exercise | 95 T1D (adol.) | HRV (time, frequency), VO_2_ | Females and non-Hispanic black adolescents had significantly lower HRV and cardiorespiratory fitness levels |
| Ang et al. (2017) [77] | ESC as a reliable surrogate for early CAN detection | Rest  CART | 37 T1D  40 C  (adults) | HRV, ESC | No significant correlations found |
| Riguetto et al. (2019) [78] | Clinical and laboratory characteristics of CAN in T1D; assess if certain clinical factors can help identify CAN | CART | 102 T1D (35% with CAN) | HRV, BP | Among other factors, hypertension, postprandial sweating, diastolic blood pressure, retinopathy, and nephropathy were considered independent predictors of CAN. |

Table S7: Summary of the articles that assessed physiological functions on the early recognition of complications other than CAN

| **Article** | **Aim** | **State** | **Cohort** | **Phys. parameters** | **Results** |
| --- | --- | --- | --- | --- | --- |
| Duvnjak et al. (2016) [79] | Cardiac autonomic dysfunction as a risk factor for diabetic retinopathy in T1D | Rest, CART | 154 T1D  (normo-albuminuric) | HRV (time, frequency), BP | The HR coefficient of variability and spectral indices of HRV might serve as a practical tool to identify a subgroup of people with T1D with higher risk of retinal deterioration |
| Pemp et al. (2018) [80] | Relationship of neuroretinal layer thickness with sensitive measures of CAN in T1D | Exercise (Orthostatic test) | 27 T1D  27C | HRV (time, frequency), BP | Correlation between inner retinal tissue loss and diminished autonomic regulation in T1D with mild-to-moderate non-proliferative diabetic retinopathy |
| Mala et al. (2017) [81] | Relationship between carotid intima media thickness and CAN in T1D | Rest,  CART | 49 T1D  45 C | HRV (time, frequency), BP | Higher carotid intima media thickness was associated with T1D and CAN. The combination of both the T1D and CAN status increased the thickness more than the sum of the individual T1D and CAN status. |
| Sekercioglu et al. (2019) [82] | Clinical and biochemical factors of diabetic kidney disease in T1D | Rest | 75 T1D  (at least 50-year-old; 25 with diabetic kidney disease) | HRV (time, frequency) | Older age at diagnosis of T1D and lower LF/HF of HRV increased the odds of having diabetic kidney disease. |
| Hansen et al. (2017)[83] | Relationship between CAN and bone metabolism in T1D | Rest, CART | 329 T1D | HR, HRV (time) | The presence of CAN was associated with reduced bone mineral density of the femoral neck (BMDfn) and increased levels of parathyroid hormone LPH. Higher resting HR associated with reduced BMDfn and increased LPH. |
| Bulum et al. (2019) [84] | Relationship between HR, systolic and diastolic BP with renal function in T1D | Rest | 313 T1D  (normo-albuminuric) | HR, BP | The urinary albumin excretion rate correlated, among other factors, with duration of T1D, (higher) HR, and diastolic BP. |
| Lu et al. (2018) [85] | Potential role of cardiovascular autonomic dysfunction in the development of renal complications in T1D | CART | 199 T1D | HRV | Enhanced CARTs, as well as BP response to standing, predicted future increases in urine albumin-creatinine ratio |
| Hotaling et al. (2016) [86] | Relationship between CAN, female sexual dysfunction, and urinary incontinence in T1D | CART | 580 T1D (women) | HRV | In long-standing T1D, CAN measurements through the Valsalva ratio and HR response to paced breathing might be a useful surrogate for generalised diabetic autonomic neuropathy and predict female sexual dysfunction |
| da Silva et al. (2019) [87] | Aggregation of cardiovascular risk factors influence on parasympathetic indices HRV in T1D | Rest | 39 T1D  (young) | HR, HRV (time, frequency), BP | In young people with T1D, the aggregation of cardiovascular risk factors was associated with parasympathetic autonomic impairment |
| Christensen et al. (2018) [88] | Prevalence of diabetic sensorimotor polyneuropathy (DSPN) and CAN in T1D and their relationship with continuous subcutaneous insulin infusion treatment | CART | 156 T1D  (young adults) | HRV (time, frequency), BP | Diabetic sensorimotor polyneuropathy and CAN were prevalent in young adults with T1D with no association found with continuous subcutaneous insulin infusion treatment |

Table S8: Summary of the articles that assessed physiological functions on the risk for long-term complications

| **Article** | **Aim** | **State** | **Cohort** | **Phys. parameters** | **Results** |
| --- | --- | --- | --- | --- | --- |
| Kim et al. (2019) [89] | Prevalence and risk factors for CAN in nonobese young people with T1D | CART | 95 T1D (adults) | HRV (time, frequency) | Higher mean HbA1c level was significantly associated with lower overall HRV |
| Jaiswal et al. (2018) [90] | Prevalence and risk factors for CAN in T1D | rest, CART | 1646 T1D  (adol., young adults) | HRV (time, frequency) | Poor long‐term glycemic, high BP were correlates of CAN in T1D |
| Guan et al. (2018) [91] | Relationship between T1D and autonomic response to acute stress | stress | 20 T1D | HRV (frequency) | People with higher HbA1c levels displayed an impaired autonomic response to stress |
| Stern et al. (2016) [92] | QTc in adolescents with T1D and associations with metabolic control and autonomic function | rest | 142 T1D  125 C (adolescents) | HR, HRV, QTc | Longer QTc was associated with higher HbA1c, lower risk of hypoglycemia and autonomic dysfunction. |
| Nyiraty et al. (2018) [93] | Relationship between autonomic neuropathy and glucose variability in T1D | CART | 20 T1D | HR | Severity of glucose variability but not overall glucose load correlates with both parasympathetic and sympathetic dysfunctions in T1D |
| Jaiswal et al. (2018) [94] | Relationship between glucose variability and non-dipping of BP as a marker of CAN in T1D | CART | 41 T1D | HRV, BP | No correlations found |
| Moser et al. (2018) [95] | Degree and direction of the HR to performance curve during cardio-pulmonary exercise | exercise | 64 T1D | degree and direction of HR | Atypical HR to performance curve during cardio-pulmonary exercise testing that were mainly related to glycemic control |
| Moser et al. (2017) [96] | Impact of glycemic control on functional capacity during cardio-pulmonary exercise in T1D | exercise | 64 T1D | HR, VO_2_ | Poor glycemic control related to less economical use of oxygen at sub-maximal work rates and an earlier time to exhaustion during cardio-pulmonary exercise. However, exercise training could have the same potential to counteract the influence of poor glycemic control on functional capacity |
